# Tapentadol: A Comprehensive Review of Its Role in Pain Management

**DOI:** 10.7759/cureus.74307

**Published:** 2024-11-23

**Authors:** Esteban Zavaleta-Monestel, Adriana Anchía-Alfaro, Jorge Villalobos-Madriz, Amil Munich, Jonathan García-Montero, Ricardo Quesada-Villaseñor, Sebastián Arguedas-Chacón, Andrés Hernández-Ortiz, Roberto Rodríguez-Miranda

**Affiliations:** 1 Pharmacy, Hospital Clínica Bíblica, San José, CRI; 2 Research, Hospital Clínica Bíblica, San José, CRI; 3 Pharmacy, Universidad Latina de Costa Rica, San José, CRI; 4 Pharmacology, Hospital Clínica Bíblica, San José, CRI; 5 Pain and Palliative Medicine, Instituto Nacional de Ciencias Médicas y Nutrición Salvador Zubirán, Mexico City, MEX; 6 Anesthesiology, Hospital Clínica Bíblica, San José, CRI

**Keywords:** analgesia, cancer, oncology pain, opioids, pain management, tapentadol

## Abstract

Pain is a prevalent issue among patients, requiring effective management to prevent the transition of acute pain into chronic pain and to mitigate significant clinical and socioeconomic impacts, such as increased morbidity, mortality, prolonged recovery, unplanned readmissions, and diminished quality of life. Despite advancements in pain management guidelines, achieving consistent pain relief remains challenging due to individual differences in pain thresholds, the nature of surgical procedures, patient age, and existing comorbidities. Tapentadol, an opioid that acts as both a μ-opioid receptor agonist and a noradrenaline reuptake inhibitor, presents a promising option for pain management. Approved by the FDA in 2008 for immediate release and in 2011 for extended release, tapentadol effectively addresses both nociceptive and neuropathic pain, offering a more favorable efficacy-safety profile compared to traditional opioids such as tramadol. Additionally, tapentadol is gaining recognition as a preferred option for managing significant pain in cancer patients due to its effectiveness and reduced side effects. This review evaluates tapentadol's clinical and pharmacological attributes, systematically analyzing literature on its efficacy, safety, pharmacokinetics, and comparative effectiveness, suggesting that tapentadol is a viable option for effective pain management with potential for broader clinical applications.

## Introduction and background

Pain management guidelines have evolved over the years to prevent easily treatable acute pain progression into chronic pain [1,2]. Despite these advancements, effective pain management remains a challenge. Several factors influence treatment decisions, including the patient's pain threshold, pain origin and location, age, and comorbidities [3]. New guidelines suggest using opioids like tapentadol to minimize patient pain and discomfort. Tapentadol, synthesized by Grünenthal Pharma in Germany and subsequently approved by the FDA in November 2008 for immediate-release (IR) and 2011 for extended-release (ER) formulations, demonstrates a favorable balance of efficacy and safety. Due to its dual mechanism of action as a μ-opioid receptor (MOR) agonist and a noradrenaline reuptake inhibitor (NRI), tapentadol is a viable option for both nociceptive and neuropathic pain [4].

Persistent postoperative pain is a common issue, with 75% of patients reporting some degree of pain after surgery [5]. Poor postoperative pain management can lead to significant clinical and socioeconomic consequences, including organ complications, increased morbidity and mortality, delayed recovery, unanticipated readmissions, reduced quality of life, and chronic postoperative pain [6]. 

Cancer-related pain is also prevalent, particularly in patients undergoing chemotherapy or radiotherapy. These treatments often exacerbate discomfort due to the immunosuppressed state of cancer patients [7]. High inflammation markers in these patients contribute to their pain, making opioids a critical component of their treatment. However, with the need for prolonged opioid use in cancer patients, new guidelines recommend tapentadol as a more refined option that still provides strong analgesia [8,9].

**Figure 1:**
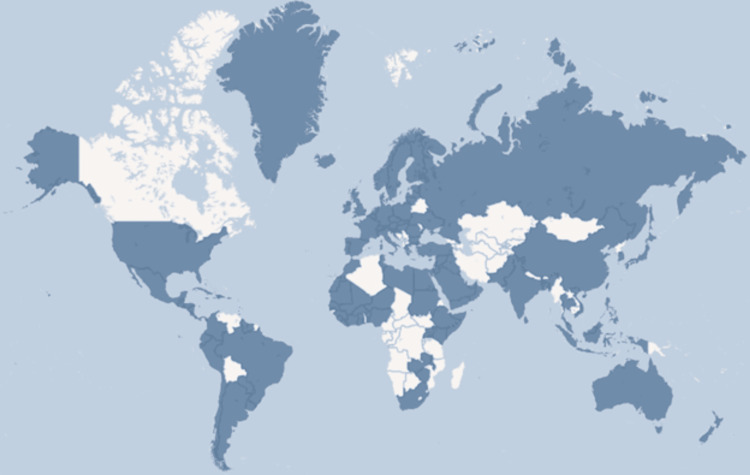
Visual representation of countries that include tapentadol in opioid class drugs for pain management Image Credit: Authors. Data visualization based on information from [12]

Tapentadol is an effective analgesic for treating moderate to severe acute pain, offering flexible treatment options. Its use as monotherapy or in combination with other analgesics ensures effective pain management, with the combination of IR and ER forms maintaining adequate serum drug concentrations. The duration of treatment and patient exposure also guide dosage and formulation choices [10,11]. Figure 1 illustrates the countries that include tapentadol in their pain management protocols [12].

This review aims to analyze the use of tapentadol in pain management, with a particular focus on cancer pain, while also examining its clinical and pharmacological characteristics.

## Review

Materials and methods

Selection Criteria and Search Strategy

A review was conducted to gather information on pain management, chronic pain, cancer pain, current medical treatment guidelines, the mechanism of action of tapentadol, its efficacy and safety profile, side effects, pharmacokinetics, and comparisons with other opioids. Molecule design was performed using tools like MarvinSketch (Chemaxon, Budapest, Hungary) to create the tapentadol structure and identify pharmacophores, while illustrations were designed using Power BI (Microsoft Corporation, Redmond, Washington, United States). The review included a collection of articles from online journals and health databases, such as Google Scholar, Clinical Key, Scopus, PubMed, and Elsevier. Keywords like "Anesthesiology guidelines", "Cancer pain", "Tapentadol", "Opioids", "Chronic pain", and "Acute pain" were used to refine the search. The primary search was conducted between May 23, 2024, and June 14, 2024.

Results

Article Selection and Study Demographics

**Figure 2:**
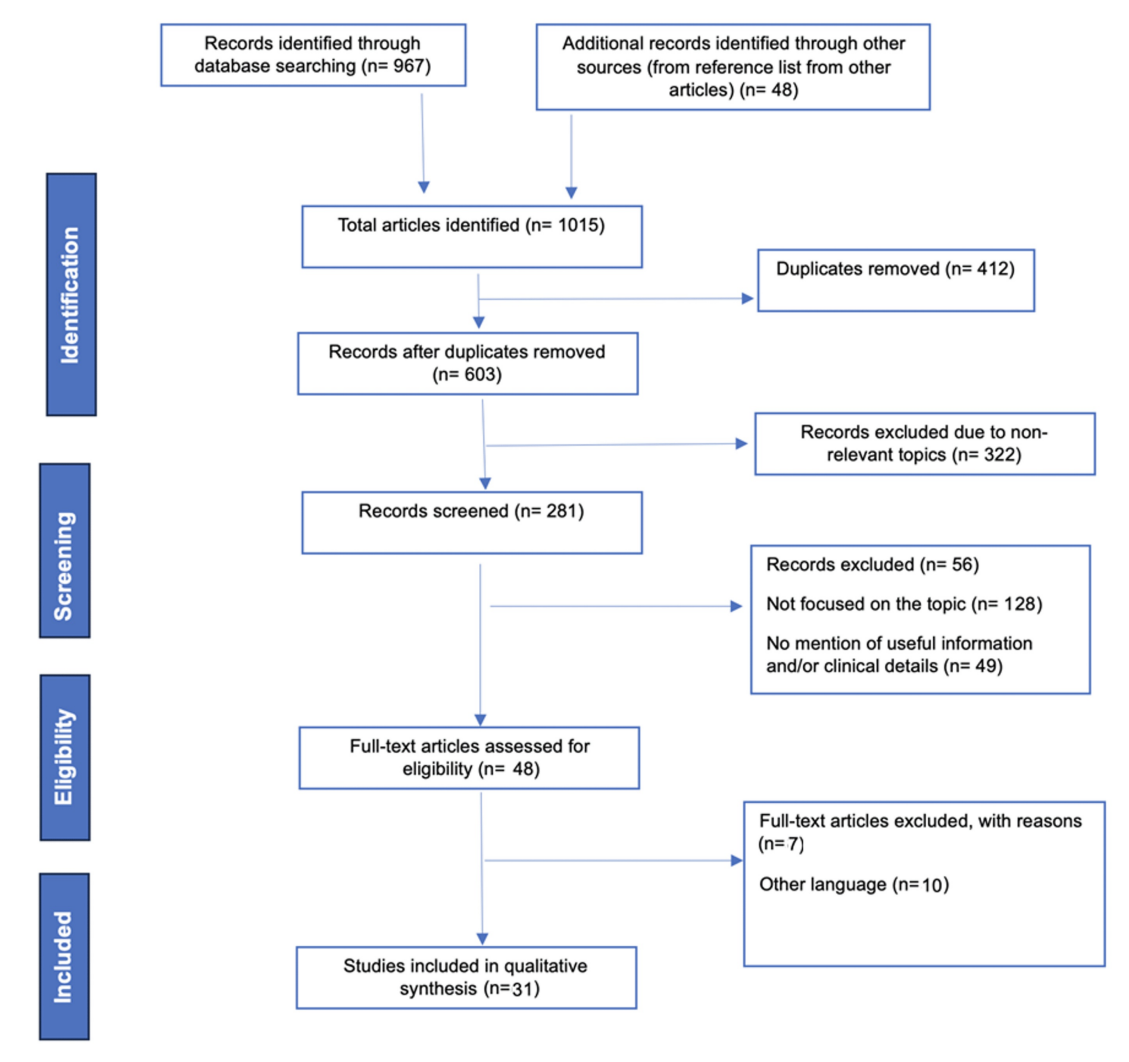
PRISMA flowchart PRISMA: Preferred Reporting Items for Systematic Reviews and Meta-Analyses Image Credit: Authors

A total of 1015 articles were found through online database searches. After removing duplicates, 603 articles were screened based on their titles and abstracts. Out of these, 322 articles were excluded due to irrelevant topics. The remaining 281 articles underwent a detailed full-text review, resulting in the exclusion of 233 articles due to insufficient useful information and clinical details. Ultimately, 48 articles were assessed for eligibility, and 31 were included in the qualitative synthesis. The Preferred Reporting Items for Systematic Reviews and Meta-Analyses (PRISMA) flowchart for the selection process is depicted in Figure 2. 

Table 1 presents the characteristics of the included studies, focusing on the clinical use of tapentadol and other opioids. A total of eight clinical trials were included to analyze the effects of opioids on the central nervous system (CNS) in humans. Articles related to cancer treatment were selected based on recent evidence supporting tapentadol over other commonly used opioids. These articles highlight tapentadol as an analgesic strategy that effectively manages both chronic and acute pain symptoms with minimal side effects. The remaining studies explored tapentadol as an effective and safe alternative to current medical guidelines on pain management, examining its pharmacokinetics and pharmacodynamics.

**Table 1:** Characteristics of the included studies, reviews, and clinical trials PR: prolonged release; CR: controlled release; ER: extended release; MOR: µ-opioid receptor; NRI: noradrenaline reuptake inhibition; IR: immediate release; OXN: oxycodone; TAP: tapentadol; RCTs: randomized controlled trials; OA: osteoarthritis; LBP: low back pain; OPO: other opioids; NP: neuropathic pain Table Credit: Authors

Author name	Title	Study design	Content summary
Afilalo and Morlion, 2013 [10]	Efficacy of tapentadol ER for managing moderate to severe chronic pain	Clinical trial	Tapentadol ER effectively treats moderate to severe osteoarthritis pain, low back pain, and diabetic neuropathy, with efficacy comparable to oxycodone CR. Its better gastrointestinal tolerability and higher patient compliance make it a potentially preferable option for long-term chronic pain management.
Roulet et al., 2021 [12]	Tapentadol versus tramadol: a narrative and comparative review of their pharmacological, efficacy and safety profiles in adult patients	Review article	Tapentadol is not a primary option for opioid therapy but serves as an additional analgesic choice, potentially beneficial for certain patients after a thorough evaluation of their clinical condition, coexisting health issues, and concurrent medications.
Schröder et al., 2010 [13]	Differential contribution of opioid and noradrenergic mechanisms of tapentadol in rat models of nociceptive and neuropathic pain	Academic article	Tapentadol is effective in treating both nociceptive and neuropathic pain. It has a superior analgesic profile compared to traditional MOR agonists. Its combined MOR-NRI activity makes it a potentially more effective option for chronic neuropathic pain.
Kress, 2010 [14]	Tapentadol and its two mechanisms of action: is there a new pharmacological class of centrally-acting analgesics on the horizon?	Academic article	Tapentadol has antinociceptive and antihyperalgesic/antiallodynic properties with a more favorable side effect profile than traditional opioids.
Riemsma et al., 2011 [15]	Systematic review of tapentadol in chronic severe pain	Systematic review	Tapentadol offers a better benefit-risk profile for chronic severe or moderate pain compared to step 3 opioids. Overall, tapentadol consistently improves tolerability across various opioids.
Raffa et al., 2012 [16]	Mechanistic and functional differentiation of tapentadol and tramadol	Review article	Tapentadol offers strong pain relief similar to oxycodone but with fewer gastrointestinal side effects.
Chang et al., 2016 [17]	Tapentadol: can it kill two birds with one stone without breaking windows?	Review article	Tapentadol produces both nociceptive and neuropathic pain relief, but with worries about abuse and dependence.
Hartrick and Rodríguez Hernandez, 2012 [18]	Tapentadol for pain: a treatment evaluation	Expert opinion	Tapentadol has shown a lower incidence of opioid-related gastrointestinal adverse effects compared to pure opioid agonists.
Jain and Basniwal, 2013 [19]	Tapentadol, a novel analgesic: review of recent trends in synthesis, related substances, analytical methods, pharmacodynamics and pharmacokinetics	Review article	Tapentadol is effective for moderate to severe acute and chronic pain. However, it is not intended for acute or mild pain and is not recommended for postoperative pain unless the patient was already on chronic opioid therapy before surgery.
Knezevic et al., 2015 [20]	Unique pharmacology of tapentadol for treating acute and chronic pain	Academic article	Tapentadol has the potential to become a uniquely suited opioid medication in the multi-modal management of moderate-to-severe acute and chronic pain conditions.
Ramaswamy et al., 2015 [21]	Tapentadol--the evidence so far	Review article	Tapentadol should not be considered a first-line opioid for moderate to severe chronic pain that can be adequately managed with other opioids. However, due to its comparable analgesic effect to modified-release oxycodone, it may be considered an alternative to morphine.
Wiffen et al., 2015 [22]	Oral tapentadol for cancer pain	Review	Pain relief and adverse events were comparable between the tapentadol and morphine and oxycodone groups.
Santos et al., 2015 [23]	Tapentadol for chronic musculoskeletal pain in adults	Systematic review	Tapentadol ER shows some pain reduction compared to placebo and oxycodone. It has a more favorable safety and tolerability profile than oxycodone.
Barbosa et al., 2016 [24]	Comparative metabolism of tramadol and tapentadol: a toxicological perspective	Review article	Tapentadol's MOR agonism offers pain relief in acute situations, while its monoamine reuptake inhibition is more effective for chronic pain. This dual mechanism enhances its versatility and allows for an "opioid-sparing" effect, providing similar pain relief at lower doses with fewer and less severe side effects compared to other opioids.
Baron et al., 2016 [25]	Effectiveness of tapentadol prolonged release (PR) compared with oxycodone/naloxone PR for the management of severe chronic low back pain with a neuropathic component: a randomized, controlled, open-label, phase 3b/4 study	Clinical trial	Tapentadol PR is noninferior and clinically superior to oxycodone/naloxone PR for severe chronic low back pain with a neuropathic component. It provided greater improvements in pain symptoms, global health, and gastrointestinal tolerability, making it a strong first-line treatment option.
Xiao et al., 2017 [26]	Efficacy and safety of tapentadol immediate release assessment in treatment of moderate to severe pain: a systematic review and meta-analysis	A systematic review and meta-analysis	Tapentadol IR 75 mg may be the most effective dose for managing moderate to severe pain while minimizing side effects. All doses of tapentadol IR are comparable in effectiveness to oxycodone HCL IR 10 mg.
Mercadante, 2017 [27]	The role of tapentadol as a strong opioid in cancer pain management: a systematic and critical review	Review article	Tapentadol, at doses equivalent to ≥60 mg of oral morphine, has proven effective and well-tolerated in opioid-tolerant cancer patients. It is a flexible option for managing moderate-to-severe cancer pain and offers the advantage of fewer gastrointestinal side effects.
Faria et al., 2018 [28]	Comparative pharmacology and toxicology of tramadol and tapentadol	Review article	Tapentadol offers several potential benefits, including lower serotonergic activity, reduced risk of dependence and abuse, more predictable pharmacokinetics, improved gastrointestinal tolerability, and suitability for treating chronic and neuropathic pain.
Pergolizzi et al., 2018 [29]	Tapentadol extended release in the treatment of severe chronic low back pain and osteoarthritis pain	Review article	Tapentadol seems to be as effective as oxycodone in treating severe chronic non-cancer pain related to LBP and OA, but with better tolerability. Its potential for abuse does not appear to exceed that of other potent opioids.
Polati et al., 2019 [30]	Tapentadol: an overview of the safety profile	Review article	Tapentadol is regarded as having a favorable long-term safety profile compared to other opioids. It demonstrates better tolerability and fewer severe side effects making it a strong choice for managing chronic pain while ensuring quality of life and patient adherence.
Morgan et al., 2019 [31]	Outcomes associated with treatment of chronic pain with tapentadol compared with morphine and oxycodone: a UK primary care observational study	Comparative study	Tapentadol PR was linked to significantly fewer gastrointestinal adverse events compared to morphine CR and oxycodone CR in patients with diagnosed pain.
Takemura et al., 2023 [32]	Differences in the analgesic effect of opioids on pain in cancer patients with spinal metastases	Comparative study	Tapentadol and methadone may be more effective than hydromorphone, oxycodone, and fentanyl for cancer pain due to spinal metastasis with numbness.
Takemura et al., 2021 [33]	Tapentadol in cancer patients with neuropathic pain: a comparison of methadone, oxycodone, fentanyl, and hydromorphone	Comparative study	Tapentadol may be effective for cancer patients with neuropathic pain and could be the preferred choice in situations requiring prompt dose adjustments or for patients at high risk of adverse effects.
Nedergaard et al., 2021 [34]	The effects of tapentadol and oxycodone on central processing of tonic pain	Clinical trial	Oxycodone and tapentadol showed analgesic effects in terms of decreased perceived pain and central processing of experimental tonic pain.
Mark et al., 2021 [35]	Although tapentadol and oxycodone both increase colonic volume, tapentadol treatment resulted in softer stools and less constipation: a mechanistic study in healthy volunteers	Clinical trial	Tapentadol treatment increased colonic volume without leading to harder stools. The results confirm that tapentadol treatment may be advantageous to oxycodone regarding tolerability to pain treatment.
Vellucci et al., 2021 [36]	Pain reduction induced by tapentadol in patients with musculoskeletal chronic pain fosters better sleep quality	Review article	The pain relief achieved with tapentadol enhances sleep quality and contributes to an improved quality of life. Thus, our results support the importance of considering sleep quality as a key outcome, alongside pain reduction, in the management of chronic pain.
Mateos et al., 2021 [37]	Long-term effectiveness and tolerability of pain treatment with tapentadol prolonged release	Clinical trial	Tapentadol PR provides ongoing pain relief and maintains quality of life for up to 72 weeks with relatively stable dosing. Its favorable safety profile suggests it is beneficial for patients with severe chronic OA knee pain and low back pain, with a limited risk of developing tolerance.
Barrachina et al., 2022 [38]	Oxycodone/naloxone versus tapentadol in real-world chronic non-cancer pain management: an observational and pharmacogenetic study	Observational study	New-generation opioids, OXN and TAP, are more effective at managing pain compared to traditional opioids, with TAP demonstrating better tolerability and reduced healthcare resource utilization compared to OXN.
Jung et al., 2022 [39]	A prospective, multicenter, open-label study of the clinical efficacy of tapentadol extended-release in the treatment of cancer-related pain and improvement in the quality of life of opioid-naïve or opioid-resistant patients	Clinical trial	Tapentadol ER effectively treats moderate to severe cancer pain and neuropathic pain, leading to significant improvements in patients' quality of life.
Barrachina et al., 2022 [40]	Sex differences in oxycodone/naloxone vs. tapentadol in chronic non-cancer pain: an observational real-world study	Clinical trial	Both the OXN and TAP groups experienced significant pain relief and fewer cases of extremely severe pain compared to the OPO group. However, the OXN group, particularly females, had poorer tolerability, a less favorable safety profile, more frequent emergency department visits for pain, and more prescription changes than the TAP group.
Boland, 2023 [41]	Tapentadol for the management of cancer pain in adults: an update	Review article	Studies have demonstrated that tapentadol is an effective analgesic, making it a viable alternative to morphine and oxycodone, particularly in cases where opioid-related toxicities are a concern.

Mechanism of action of tapentadol

Tapentadol exerts a synergistic analgesic effect through its dual action as a MOR agonist and a NRI, targeting both the ascending and descending pain pathways. While MOR inhibition alone may have limited efficacy in chronic pain, tapentadol's effectiveness is also driven by its ability to inhibit noradrenaline (NA) reuptake. This inhibition increases extracellular NA concentrations in the spinal cord, leading to an antinociceptive effect via the activation of α2-adrenoceptors (A2-AR) [13].

**Figure 3:**
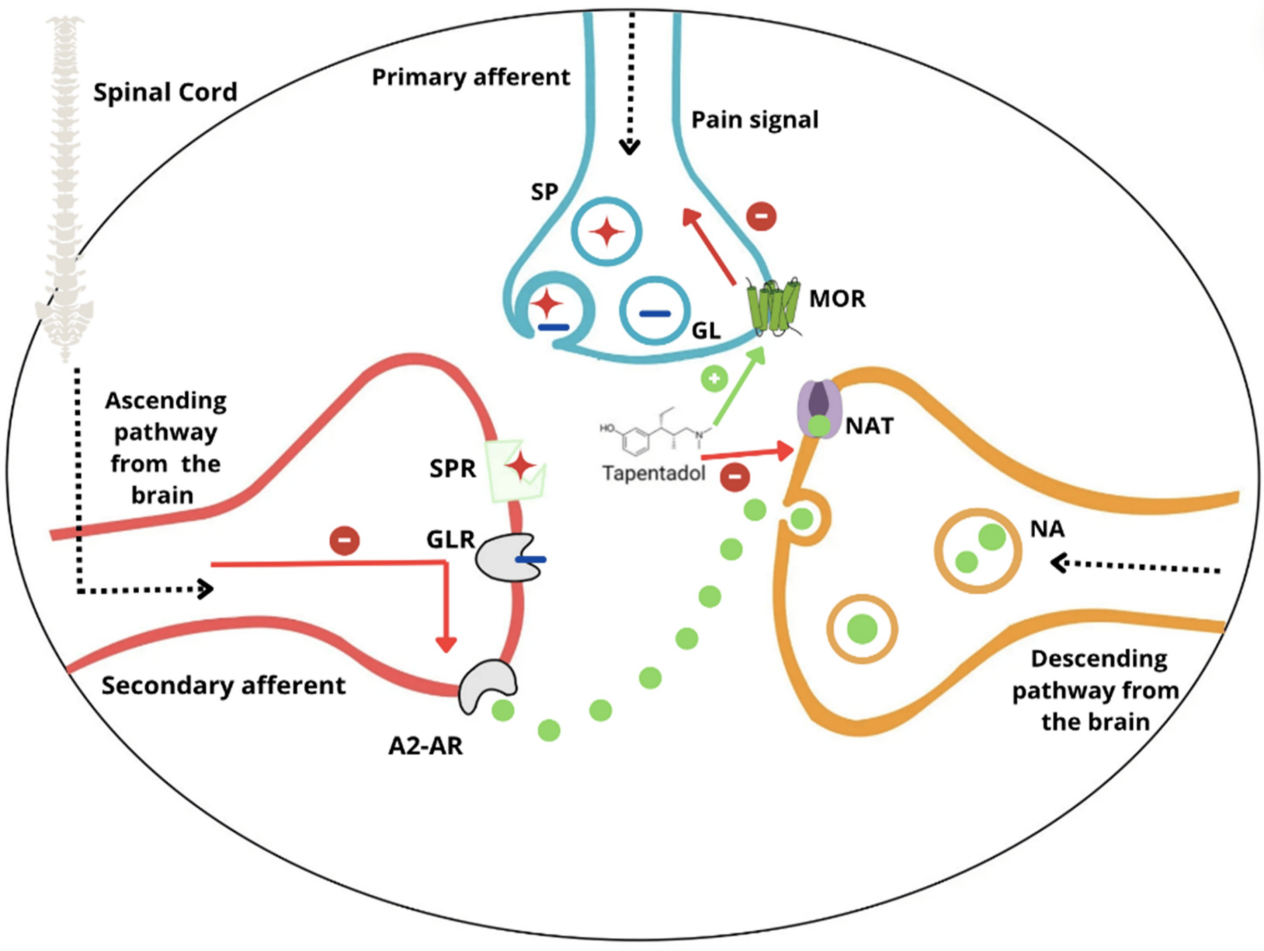
Tapentadol mechanism of action. Employing its effect on MOR and as a noradrenaline reuptake inhibitor. Also acting on A2-AR, SPR, and GLR SP: substance P; GL: glutamate; MOR: µ-opioid receptor; SPR: substance P receptor; GLR: glutamate receptor; A2-AR: alpha 2-adrenoreceptor; NA: noradrenaline; NAT: noradrenaline transporter Image Credit: Alshehri [42]. Originally published by, adapted, and used with permission from Dove Medical Press Ltd.

Additionally, tapentadol's analgesic effect is enhanced through its influence on the serotonin (5-HT) reuptake pathway. 5-HT is a key neurotransmitter in descending pain pathways from the brainstem to the spinal cord, and when its reuptake is inhibited, 5-HT receptors on spinal neurons are more actively engaged, modulating spinal nociceptive signaling. This combined effect reduces the release of glutamate (GL) by neurons, thereby decreasing the transmission of pain signals to the CNS and achieving an overall analgesic effect [14]. Figure 3 illustrates the mechanism of action of tapentadol in the ascending and descending pain pathways.

Structure optimization and pharmacophores

The primary pharmacophore of this opioid is the diethylaminomethyl portion of the molecule, which is essential for its biological activity and crucial interactions with MOR and NA receptors. Compared to tramadol, the cyclic ring system in tapentadol has been modified by enlarging the phenyl ring and incorporating a hetero atom, such as oxygen. These modifications significantly enhance the molecule's potency. The enlarged phenyl ring improves binding energy, leading to greater flexibility and ligand efficiency, while the added hydrogens contribute to increased physicochemical stability and sustained analgesic activity [42].

Pharmacodynamics

This drug's dual mechanism of action supports its effectiveness against a wide range of pain types, including both chronic and acute pain. It works through MOR agonist activity in the ascending pain pathways within the spinal cord, peripheral tissues, and brain while also enhancing descending pain modulation via NRI [14,17]. Although tapentadol is less potent than morphine, this may be attributed to its NRI-mediated opioid-sparing effect, making it about three times less analgesic. Its lower binding affinity for MORs compared to morphine further explains its reduced potency. However, this lower affinity also contributes to tapentadol's reduced side effect profile relative to traditional opioids. The drug's ability to interact with both NA and 5-HT pathways enhances its efficacy in treating chronic and acute pain and creates a synergistic effect when used with morphine, a potent MOR activator. This combination allows for effective pain management while mitigating the side effects commonly associated with classic opioids. This binding activity also creates a synergistic effect between tapentadol and morphine [19,20].

Pharmacokinetics and metabolism

Tapentadol has demonstrated an oral absorption rate of approximately 30%, which is relatively low. Its pharmacokinetic profile follows a linear model, and its absorption is not significantly affected by food intake, gastric pH, or gastrointestinal motility [19]. Unlike some other opioids, tapentadol is not a prodrug, allowing for rapid distribution and absorption without the need for metabolic activation. Following oral administration, tapentadol reaches its peak serum concentration in about 1.5 hours [19,43].

Approximately 97.3% of tapentadol undergoes first-pass hepatic metabolism, which contributes to its low interaction with other medications, as it has minimal involvement in phase I metabolic pathways. Unlike opioids like tramadol, tapentadol's metabolism involves deactivation without the formation of active metabolites, and its analgesic effect lasts for about six hours after oral administration [43].

Tapentadol's metabolism involves minimal mediation by the cytochrome P450 system, relying primarily on phase II pathways, particularly glucuronidation. The drug is metabolized in the liver by enzymes such as CYP450, CYP2C9, CYP2C19, and CYP2D6, with approximately 99% of its metabolites excreted via the kidneys. Due to this, caution and close monitoring are necessary when administering tapentadol to patients with compromised renal function [42,43].

Tapentadol's pharmacokinetic profile is generally considered safe and effective for patients undergoing multiple treatments. However, its efficacy can be influenced by the polymorphic enzyme CYP2D6, resulting in variable and sometimes unpredictable analgesic effects across different genetic backgrounds [42].

In direct comparisons with other opioids, tapentadol shows a superior efficacy-to-safety ratio and lower rates of adverse effects compared to traditional opioids [34,44]. For instance, while postoperative pain often requires the use of synthetic opioids, the associated adverse effects can impede recovery [31]. One of tapentadol's key advantages is its minimal impact on the serotonergic system, as its metabolic activation bypasses the CYP450 system. This is particularly important because activation of the CYP450 system, prevalent in the metabolic pathways of many opioids, frequently leads to drug-drug interactions with other medications that utilize the same pathway [45].

The most undesirable adverse reactions associated with opioids stem from their effects on the monoaminergic system [35]. The monoaminergic system is crucial for maintaining basic functions across the human body, and its disruption can negatively affect multiple organs, including the brain, stomach, heart, lungs, and reproductive organs. This can result in a range of side effects, such as respiratory depression, nausea, vomiting, bowel dysfunction, constipation, heart palpitations, anxiety, and physical dependence [28,37,46].

Tapentadol's lower impact on the norepinephrine MOR also explains its reduced effect on the cardiovascular system [30]. This mechanism further accounts for its minimal influence on androgen levels. A study found that concentrations of testosterone, luteinizing hormone (LH), and follicle-stimulating hormone (FSH) in patients taking tapentadol were similar to those in the placebo group and significantly lower than in patients administered oxycodone or morphine [47].

When comparing the potency of various opioids to morphine, which is considered the gold standard in opioid treatment, tapentadol demonstrates a similar potency to oxycodone and tramadol while minimizing major side effects and withdrawal symptoms. This comparison is made using a conversion factor unique to each opioid, based on its physicochemical properties. Understanding these conversion factors is essential for accurately assessing opioid potency and dosage. Table 2 presents the equianalgesic doses of opioids, equivalent to 10 mg of morphine [17].

**Table 2:** Practical equianalgesic doses of opioids, equivalent to 10 mg of morphine Table Credit: Authors. The values presented in this table were calculated by the authors using data from [17]

Opioid	Practical equianalgesic dose (mg)	Conversion factor	Equivalent to 10 mg of morphine
Tapentadol	25	÷3	8.3 mg
Oxycodone	5-7.5	×1.5	9.5 mg
Codeine	75-90	÷8	10.3 mg
Tramadol	50	÷5	10 mg

Tapentadol in cancer pain management

When treating cancer, studies indicate that over 40% of patients experience some degree of pain, ranging from moderate to severe. This is a critical factor to consider in cancer treatment, as it significantly impacts patients' quality of life and their resilience to cancer therapies, including the side effects of chemotherapy [39]. Managing cancer pain is particularly challenging due to its classification as both nociceptive and neuropathic. The World Health Organization (WHO) recommends a stepwise approach to pain management in cancer patients, beginning with weaker opioids and progressing to stronger ones. However, some healthcare practitioners advocate starting with more potent opioids for more effective pain relief [24,39].

Tapentadol is a viable option for cancer pain management due to its dual mechanism of action, which provides synergistic analgesia for both nociceptive and neuropathic pain [48]. It stands out as a preferable choice for cancer patients because of its reduced side effect profile compared to other opioids. Given the numerous side effects associated with radiotherapy and chemotherapy, the addition of another medication to manage pain must be carefully considered to avoid exacerbating adverse events [49].

Tapentadol offers advantages over other opioids due to its flexible dosing regimens, good tolerability, and efficacy. Clinical evidence suggests that long-term treatment is often necessary for cancer patients, making safety profiles and toxicological properties crucial factors in selecting an analgesic [41]. Notably, tapentadol has been shown to cause minimal to no physical dependency, even with prolonged use. This is an essential consideration, as many opioids are associated with withdrawal symptoms due to their effects on the monoaminergic system [27]. Moreover, tapentadol's metabolism bypasses the CYP450 pathway, resulting in fewer drug-drug interactions, making it a suitable option for cancer patients undergoing concurrent treatments [33].

While many analgesics aim to replicate the effects of morphine, it is essential to consider their long-term impact on organs. A study found that tapentadol provided pain relief in 93% of opioid-naïve and opioid-tolerant patients within four days of treatment, with doses ranging from 50-100 mg/day to 50-400 mg/day [50]. This evidence underscores tapentadol's effectiveness and safety as an analgesic for cancer pain management.

## Conclusions

The reviewed studies indicate that tapentadol is an effective and safe analgesic for managing moderate to severe acute pain. Tapentadol stands out among traditional opioids due to its dual mechanism of action, reduced side effects, improved tolerability, and minimal drug-drug interactions. These features make it a promising option for pain management and cancer-related pain management, as clinical trials have consistently demonstrated its efficacy and favorable safety profile in these contexts. Future research should focus on the long-term efficacy and safety of tapentadol in chronic pain management and explore the potential benefits of combination therapies with other analgesics.
